# Three Cases in General Surgery in Which the Dental Engine Was Employed for Exsection of Bone

**Published:** 1882-02

**Authors:** J. S. Wight


### THREE CASES IN GENERAL SURGERY IN WHICH THE DENTAL ENGINE WAS EMPLOYED FOR EXSECTION OF BONE.

Case i.—A boy about eighteen months of age had empyema of the left side of the thorax; a patient of Dr. Harrigan. On consultation it was determined to open the pleural cavity and let out the pus. I recommended the use of the dental trephine as the best means of getting through the rib. Dr. B. F. Westbrook gave the patient chloroform, and assisted by Dr. Harrigan I performed the following operation: An incision down on the eighth rib, two inches in length, was made, about midway between the sternum and spine; a small portion of the periosteum was raised from the rib; a trephine three-sixteenths of an inch in diameter was applied to the upper half of the rib, and by means of the dental engine a small button of bone was cut out and removed in a very few seconds; the same trephine was then applied to the lower half of the same rib, backward and downward from the place of its first application, and another button of bone was very quickly removed; there was then a complete solution of the continuity of the rib; the posterior end of the anterior costal fragment was gently raised by a small elevator, and a minute piece of it cut off with a pair of curved bone-pliers; the anterior end of the posterior costal fragment was also raised in the same manner, and a minute piece of it cut off with the same bone-pliers; there was then an opening down to the subcostal tissues, about three-eighths of an inch wide and about six-eighths of an inch long; in this opening, with a curved bistoury, a transverse incision about four-eighths of an inch in length was made directly into the pleural cavity; then there was slowly evacuated about one-half pint of so called healthy pus. During the entire operation there was but very little bleeding, the intercostal artery being avoided. In fine, I am quite sure that the operation in this case was performed more speedily and safely by the means employed than it could have been by any other means at our command. And it may be of interest to know that this case did well after the operation, which was performed on the 25th day of April, 1881.

CASE 2.—May 1st, 1881. Mary S., twenty months of age, was trampled on by a vicious horse, and had one of her ribs on the left side broken, near the sternum, and had a compound depressed fracture of the skull on the right side, about three and one-half inches directly above the meatus auditorius. The line of fracture was crescentic in form, and about two inches in length. A large piece of bone was bent downward, about three-sixteenths of an inch; the patient was unconscious and the pupils were moderately dilated; there was marked left hemi-spasm, especially of the upper extremity; there was copious bleeding from the nose, and there was some vomiting of blood, and it was decided, on consultation with Drs. Matheson and Northridge, to elevate the depressed bone.

Dr. Matheson made an incision over the edge of the sound bone and the depressed edge of the broken bone, and cleared away a small portion of the periosteum, so that I could apply my dental trephine at the edge of the sound bone. The trephine was one-fourth of an inch in diameter, and with a few turns of the wheel of the dental engine, in a few seconds, a small button of bone had been removed, without any injury whatever to the dura mater. Then Dr. Matheson gently inserted a small, strong elevator into the trephine hole, and under the edge of the depressed bone, using the sound bone of the skull for a fulcrum, and with the employment of considerable force, raised up the edge of the depressed fragment. The entire operation occupied only a few moments, and I am confident did not add materially to the severity of the shock ; certainly the trephining could not have been done so speedily, and with so little disturbance, if I had used the ordinary instrument. It may be a matter of interest to note that this little patient did not survive her injuries.

And it may be remarked that in this case the advantages of the dental trephine may be stated by saying, there was the minimum of shock from the operation, because it was done quickly and safely, and without any special or marked additional disturbance of the general system. In fact, it is somewhat difficult to trephine a very thin skull with the ordinary trephine, as I know by experience, and so I incline to the view that the dental trephine will be found to be more manageable than the ordinary trephine in operating on a very thin skull.

CASE 3.—A man about fifty years of age, a patient of Dr. Wallace, had severe inflammation in and around the right knee joint, ending in peri-articular abscess and some necrosis of the upper end of the tibia. This patient was admitted to the Long Island College Hospital, where in the presence of the medical class, I made two incisions just below the knee joint, one on each side of the tubercle of the tibia, down to the bone. The periosteum at the bottom of each incision was carefully raised up, when a dental trephine one-fourth of an inch in diameter was applied, and a button of bone was removed from the compact tissue on either side. This permitted an examination of the cancellous bone in the base of the tibia, which was found to be necrosed quite extensively. The openings already made were now enlarged by a gouge and pliers, and the necrosed bone was extensively dug and scraped out, leaving, for the purpose of support, a firm bridge of compact bone in front. The cavity was dressed by packing it with oakum containing carbolized oil, and it quite rapidly filled in with scar-tissue. There was no special surgical fever following the operation. In this case Esmarch's bandage was used, rendering the operation bloodless. Also it may be noted that the operation was done under the carbolic spray ; and it is worthy of remark that there was no fresh disturbance set up in the joint, notwithstanding the layer of bone left across the base of the tibia was very thin.

The above cases incline me to believe that the dental engine will be a valuable addition to the instruments of the surgeon. I am convinced that the surgeon can do some pieces of work better with the dental engine than he can with the usual instruments. Small and short points of broken bone can be safely and quickly cut off with a small circular saw run by the dental engine, and holes can be made with accuracy, facility and safety, in the ends of resected bones, for the purpose of inserting wire sutures. This would have an advantage over the ordinary hand drill, which is liable to slip and cause damage to the tissues. In one instance, swiftness of motion is employed; in the other, considerable pressure is used to perforate the bone. In resecting small ribs and thin skulls, and in exploring the ends of the long bones, the dental engine will no doubt prove to be of unquestionable utility, because it is safe, accurate and speedy.—Prof. J. S. Wight, in *Med. and Surg. Reporter.*

